# Advances in Supercritical Carbon Dioxide Extraction of Bioactive Substances from Different Parts of *Ginkgo biloba* L.

**DOI:** 10.3390/molecules26134011

**Published:** 2021-06-30

**Authors:** Ruihong Li, Ziming Xia, Bin Li, Ying Tian, Guangjie Zhang, Min Li, Junxing Dong

**Affiliations:** 1 School of Pharmacy, Henan University, Kaifeng 475000, China; lrh1217021@163.com; 2 Department of Pharmaceutical Sciences, Beijing Institute of Radiation Medicine, Beijing 100850, China; naisixx@163.com (Z.X.); jkylibin@hotmail.com (B.L.); hq6106@aliyun.com (Y.T.); zhanggj410@sina.com (G.Z.)

**Keywords:** *Ginkgo biloba* L., active ingredients, supercritical CO_2_ fluid extraction

## Abstract

*Ginkgo biloba* L. has always been a popular area of research due to its various active ingredients and pharmacological effects. *Ginkgo biloba* is rich in ginkgo flavonoids, ginkgolides, and ginkgolic acid, with anti-inflammation, antioxidation, neuroprotection, anti-platelet agglutination, hypolipidemic effect, anti-cancer, and anti-radiation properties. There are many methods to extract and separate the active components of ginkgo. Among them, supercritical carbon dioxide fluid extraction (SFE-CO_2_) is known for its green, clean, and environment-friendly properties. In this paper, the pharmacological activities, the active components, and structures of different parts of ginkgo, the extraction methods of its effective ingredients, and the application of the SFE-CO_2_ method for the extraction and separation of active ingredients in *Ginkgo biloba* from leaves, seeds, pollen, and roots were reviewed, in order to make best use of ginkgo resources, and provide support and references for the development of SFE-CO_2_ of active components from *Ginkgo biloba*.

## 1. Introduction

*Ginkgo biloba* L., also called maidenhair tree, is a rare species of Mesozoic relict and the sole species in the class Ginkgopsida, also known as the “living fossil” [1,2]. *Ginkgo biloba* is rich in a variety of natural active ingredients and has a wide range of pharmacological activities [3], which plays an important role in food [4,5], health care [6], medicine [7,8], and other fields. The traditional methods of extracting active substances from *Ginkgo biloba* include solvent extraction [9], enzymatic hydrolysis [10], and resin extraction [10]. Compared with supercritical carbon dioxide fluid extraction (SFE-CO_2_), these methods have many disadvantages, such as low yield, the excessive residue of harmful substances, and long extraction. As a new extraction and separation method, SFE-CO_2_ has aroused increasing concern due to less use of organic solvents, short extraction time, and high yield. In recent years, several studies [11,12] also showed that the yield of bioactive components in *Ginkgo biloba* will be higher if SFE-CO_2_ is applied to its extraction and separation. However, no review is available on the SFE-CO_2_ of active components from different parts of *Ginkgo biloba*. Therefore, this paper reviewed the biological activities of different parts of *Ginkgo biloba* and the application of SFE-CO_2_ in different parts of ginkgo, attempting to provide support and reference for the further development of SFE-CO_2_ technology of active components from *Ginkgo biloba.*

## 2. Biological Activity of *Ginkgo biloba*

**Figure 1:**
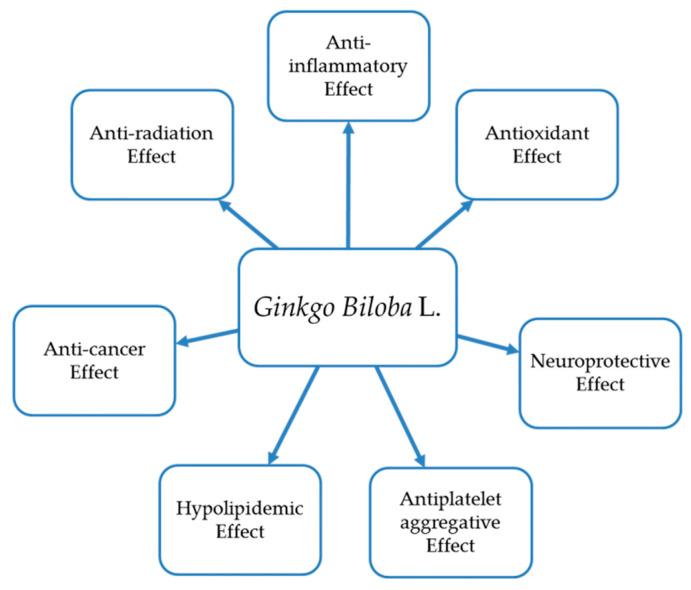
Bioactivity of *Ginkgo biloba* L.

Modern pharmacological studies revealed the various biological activities of ginkgo, such as anti-inflammatory, antioxidation, neuron protection, anti-platelet aggregation, hypolipidemic effects, anti-cancer, anti-radiation properties, etc. The main biological activities of *Ginkgo biloba* are as follows (Figure 1):

### 2.1. Anti-Inflammatory Effect

Li et al. [13] found that the ethyl acetate and chloroform parts of the ginkgo male flower extract could significantly downregulate inflammatory cytokines of nitric oxide (NO), TNF-α, IL-6, PEG_2_, iNOS mRNA, and COX-2 mRNA in a dose-dependent manner, which made them the anti-inflammatory active parts of ginkgo male flowers. Li et al. [14] found that ginkgolide A could reduce the inflammatory response induced by lipopolysaccharide in vivo and in vitro. Zhang et al. [15] found that *Ginkgo biloba* extract EGB761 could inhibit inflammation by regulating inflammatory cytokines and mediators in vivo and in vitro. The main active ingredient flavonoid glycosides could inhibit the release of inflammatory cytokines in LPS induced RAW264.7 macrophages.

### 2.2. Antioxidant Effect

Ginkgo extract is rich in flavonoids, which could be used as a natural antioxidant. It could play an antioxidant activity by scavenging free radicals, superoxide anions, and NO, as well as inhibit free radical reaction and lipid peroxidation [16,17]. Martínez-Solís et al. [18] found that flavonoids and terpenoids in ginkgo leaf extracts could be used to prevent and treat retinal diseases related to oxidative stress. Liu et al. [19] found the activities of superoxide dismutase (SOD) and glutathione peroxidase (GSH-Px) could be improved by ginkgolide while the concentration of malondialdehyde (MDA) in the myocardial tissue of myocardial ischemia-reperfusion (MI/R) rats. Ren et al. [20] isolated polysaccharide compounds GBPS-2 and GBPS-3 from ginkgo leaves, which could significantly scavenge superoxide radicals and 3-ethylbenzothiazoline-6-sulphonic (ABTS) radicals, as well as hydroxyl free radicals and DPPH free radicals.

### 2.3. Neuroprotective Effect

Ginkgolide B could protect the nervous system by inhibiting platelet-activating factors, reducing the concentration of calcium ions, and downregulating the level of NO [16]. Meanwhile, ginkgo leaf extract could also enhance the local cerebral blood flow to reduce cerebral ischemia and hypoxia [17]. The accumulated amyloid β protein (Aβ) in the brain is a key feature in Alzheimer’s disease. Sasaki et al. [21] found that kayaflavone and sciadopitysin showed a higher inhibitory effect on Aβ42 cytotoxicity in rat adrenal pheochromocytoma (PC12) culture. Li et al. [22] found that ginkgo diterpenoid lactones could reduce the cerebral infarction area and improve the neurological deficit and brain edema remarkably. Kaur et al. [23] investigated the protective effect of *Ginkgo biloba* leaf extract (GBE) on hippocampal neuronal injury induced by methyl tin chloride (TMT) and suggested that GBE can protect hippocampal neuronals by downregulating the NO level.

### 2.4. Antiplatelet Aggregative Effect

Platelet-activating factor (PAF) could induce platelet aggregation and lead to cardiovascular disease [16]. Ginkgolides are the primary active substances in ginkgo that inhibit platelet aggregation. Xu et al. [24] used PAF as an inducer of platelet aggregation, and added ginkgolide A (GA), ginkgolide B (GB), ginkgolide C (GC), ginkgolide J (GJ), ginkgolide K (GK), and ginkgolide M (GM) to the rabbit blood, respectively, to compare the antagonistic effects of different compounds on platelet aggregation. It was found that the above-mentioned ginkgolides had certain antagonistic effects on platelet aggregation, and the activities of GB and GK were the strongest in a dose-dependent manner. Wang et al. [25] reported that *Ginkgo biloba* extract could exert its anti-platelet aggregation, antioxidation, and hypolipidemic effects through comprehensive regulation of multiple metabolic pathways, to prevent and treat myocardial infarction.

### 2.5. Hypolipidemic Effect

Chen et al. [26] found that *Ginkgo biloba* extract could reduce the accumulation of cholesterol in peripheral tissues, lessen the damage of vascular endothelial cells, and resist the production of atherosclerosis. Chen et al. [27] showed that ginkgo leaf extract (GBE) possessed a significant hypolipidemic effect. Compared with the hyperlipidemia rat model group, the middle and high-dose GBE groups had the lower total cholesterol, low-density lipoprotein cholesterol, and triglyceride in serum, while their high-density lipoprotein cholesterol was significantly increased, which proved that *Ginkgo biloba* has an obvious effect on reducing blood lipids. Feng et al. [28] reported that 66 patients with hyperlipidemia were treated with ginkgo leaf extract. The results showed that their microcirculation disorders were improved and the hemorheological abnormalities and blood lipid levels were effectively regulated.

### 2.6. Anti-Cancer Effect

The bioflavonoids in male flowers of *Ginkgo biloba* were demonstrated to exhibited anti-cancer effects. Among them, bilobetin and isoginkgetin were capable of arresting the cell cycle in the G2/M phase, inducing the apoptosis-associated protein to specifically inhibit the proliferation of HeLa cells [29]. Fu et al. [30] explored the anti-cancer activity of GBE in vitro on human gastric cancer cells SGC-7901 and MGC-803 and found that GBE could inhibit the metastasis of gastric cancer in a dose-dependent manner. Ma et al. [31] reported that ginkgolic acid in ginkgo exopleura could inhibit the growth of pancreatic tumor cells by downregulating the expression of key enzymes in adipogenesis, such as acetyl CoA carboxylase and fatty acid synthase.

### 2.7. Anti-Radiation Effect

During World War II, the U.S. military dropped the first atomic bomb on Hiroshima, Japan. Within five kilometers of the explosion, buildings were flattened and no grass was left. One month later, the ginkgo tree sprouted at a distance of one kilometer from the nuclear explosion center, which was of great significance for our study on the radiation resistance of ginkgo. After the Chernobyl nuclear power plant accident in 1986, Emerit found that the chromosome breakage factors appeared in the blood of survivors. This factor decreased or disappeared in the plasma of survivors two months after EGB761 administration, proving the anti-radiation effect of *Ginkgo biloba* extract [32]. Shi et al. [33] suggested that GBE was able to reduce the micronucleus rate of bone marrow polychromatic erythrocytes and the chromosome aberration rate of bone marrow cells in mice by X-ray radiation. Li et al. [29] compared the anti-radiation effects of ginkgo leaves and ginkgo flowers extract on the irradiated C57BL/6J mice with ^60^Co γ rays. They demonstrated that both ginkgo leaves and ginkgo flowers extract could prolong the average survival time of irradiated mice, but ginkgo flowers performed better and significantly promoted the recovery of peripheral blood cell level of irradiated mice.

## 3. The Active Components and Structure of Different Parts of *Ginkgo biloba*

**Figure 2:**
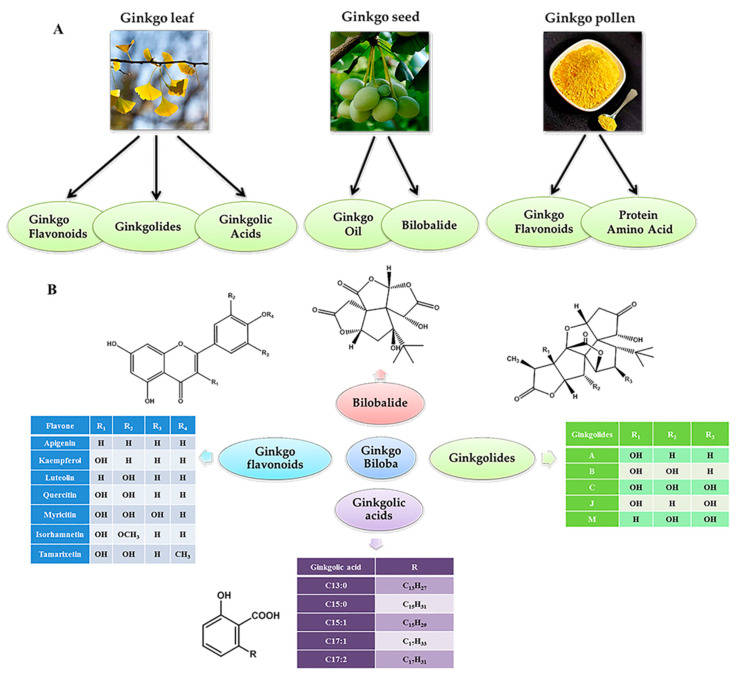
The active components (**A**) and related structures (**B**) in *Ginkgo* *biloba* L.

The components of ginkgo varies with different parts of it, such as ginkgo leaves, seeds, pollen, branch bark, and root bark (Figure 2A). The main active ingredients of ginkgo leaves include ginkgo flavonoids, ginkgolides, and ginkgolic acids [34]. Ginkgo seeds contain ginkgo oil, *bilobalide*, etc. [35]. Similar to ginkgo leaves, ginkgo pollen is rich in ginkgo flavonoids [35]. In addition, the amino acids, proteins, and other substances in ginkgo pollen are also abundant.

As shown in Figure 2B, flavonoids are the compounds with the parent structure of 2-phenylchromone and no substitution of oxygen-containing groups at the 3-position [36]. Ginkgo flavonoids usually have substituents at the 3-position of the C ring, 3′-position, 4′-position, and 5′-position of the B ring, such as apigenin, kaempferol, luteolin, quercetin, etc. At present, more than 70 kinds of flavonoid structures have been isolated and identified from *Ginkgo biloba*, of which nearly half are from *Ginkgo biloba* leaves [37]. The main types of these structures include flavonoids (alcohols) and their glycosides, dihydroxyflavones, biflavonoids, and catechins. Ginkgolides are compounds belonging to terpenoids, which are composed of sesquiterpene lactones and diterpene lactones. Ginkgolide A, ginkgolide B, ginkgolide C, ginkgolide J, ginkgolide M, and some other ginkgolides have been discovered from ginkgo at present. Bilobalide is the only half terpene compound from ginkgo. Ginkgolic acids were the lacquer phenolic substances, which were the secondary metabolic products of ginkgo [38]. Ginkgolic acids include simple phenolic acids and alkyl phenolic acids. Beek et al. [39] isolated ginkgolic acids with six substituents C13:0, C15:0, C15:1, C17:1, C17:2, and C17:3 from ginkgo leaves by liquid phase preparation method.

At present, there are only a few studies on the components in branch bark and root bark of ginkgo using solvent extraction method [40,41,42]. Studies have shown that ginkgo root contains a large number of flavonoids mainly composed of quercetin, and does not contain kaempferol, isorhamnetin, and other substances [40]. Su et al. [41] separated bilobalide, ginkgolide B, ginkgolide C, some fatty alcohols, and fatty acids from ginkgo bark by organic solvent extraction. Importantly, protocatechuic acid, vanillic acid, and daucosterol were isolated from ginkgo for the first time. Zhao et al. [42] used the organic solvent extraction method to extract 13 compounds from ginkgo root bark, among which stearic acid, behenic acid, and lignoceric acid were isolated from ginkgo for the first time.

## 4. The Practical Significance on SFE-CO_2_ of *Ginkgo biloba* Bioactive Components

### 4.1. Supercritical CO_2_ Fluid Technology

Supercritical fluid (SF or SCF) is the fluid that exceeds the critical temperature (Tc) and critical pressure (Pc). The fluid in this state has no obvious gas–liquid boundary and has the characteristics of low viscosity, high density, and high diffusivity [43]. Compared with liquid and gas, supercritical fluid has obvious advantages in viscosity, density, and diffusion coefficient. Its density is close to that of liquid. Viscosity is close to that of gas, and the diffusion coefficient is 10~100 times of liquid [44]. Therefore, supercritical fluid could be used as an excellent solvent for the extraction and separation of substances. Common supercritical fluids include carbon dioxide (CO_2_), water, ethylene, ethane, butane, methanol, etc. [45]. As a supercritical fluid, CO_2_ has a critical temperature of 31.1 °C and a critical pressure of 7.38 MPa. Its operating temperature and pressure are relatively easy to achieve [44]. CO_2_ is one of the most frequently used solvents in supercritical fluid extraction (SFE), with the advantages of reaching the supercritical fluid state readily, safety, non-toxicity, non-flammability, and high elimination rate [46].

Due to its high density and low viscosity, SFE has excellent solubility and permeability for many substances. Extractive selectivity could be achieved by changing the temperature, pressure, and co-solvent. The extract is easily recovered by simple decompression, allowing the supercritical CO_2_ to return to the gaseous state and evaporate, leaving little or no trace of the solvent [47]. When supercritical CO_2_ fluid is used as the solvent to extract the active components of plants, the CO_2_ contacts with the substances to be separated and dissolves the components in turn, according to the boiling point, polarity, and molecular weight. When the CO_2_ and dissolved solute reach the separation kettle, the separation kettle that deviates from the supercritical region could make the solute and CO_2_ separated rapidly, the solute sedimentation forms the extract, and the CO_2_ could recover its dissolution ability after the heat exchanger, to achieve the purpose of extraction and separation [48].

The easy scale-up procedure for SFE processes consists of two steps [49]. The first is to perform small scale assays to define the optimal extraction conditions through a screening of operational parameters. The second step involves the selection of the scale-up method based on the kinetic limiting factors. There are also several mass transfer models to explain the extraction curves, among which, the logistic model, the diffusion model, and Sovová model are the most convenient [50].

### 4.2. Study on Extraction of Natural Products by SFE-CO_2_ Technology

The decaffeination of raw coffee by supercritical CO_2_ fluid technology was studied in 1970 by Kurt Zosel at the Max Planck Institute, which was considered the first technical application of this separation technology [51]. Later, the study on the extraction of other natural products by SFE-CO_2_ technology was reported successively in several papers [52,53]. To design a reasonable SFE process, it is necessary to understand the mass transfer mechanism and appropriate mathematical representation of the extraction process. Zhen et al. [54] reviewed the mass transfer mechanism of the extraction process and the appropriate mathematical representation under the design of SFE. Mechanisms involved in a mass transfer model are discussed in terms of external mass transfer resistance, internal mass transfer resistance, solute–solid interactions, and axial dispersion, which contributed to employing for modeling SFE of natural matters.

Similarly, factors, such as flow rate and residence time of CO_2_, could affect the extraction of natural products. Wilkinson et al. [55] indicated that a residence time of less than 60 s for the SFE-CO_2_ would be sufficient for complete the extractions of soybean oil by the exploration of flow rate.

Compared with other extraction methods, SFE-CO_2_ could effectively improve the extraction rate. Yu et al. [56] compared different extraction methods for the volatile oil from Torreya Grandis aril. He found that, compared with the organic solvent extraction method (18.28 ± 0.14%) and steam distillation (2.17 ± 0. 02%), the rate of SFE-CO_2_ was the highest (22.12 ± 0.09%). Yan et al. [57] found that the yield of *Ginkgo biloba* extract reached 2.1% under the conditions of extraction pressure of 30 MPa, the temperature of 60 °C, and the concentration of co-solvent of 5% ethanol. The yield of this method was 1.8% higher than that of the traditional extraction method, and the contents of total flavonoids and terpenoids were also significantly improved.

At present, SFE-CO_2_ technology was applied to extract essential oil, terpenoids, alkaloids, flavonoids, anthraquinones, phenylpropanoids, saponins, polysaccharides, and other active components of traditional Chinese medicine [58].

Various methods have been applied to the extraction of active ingredients from *Ginkgo biloba* including solvent extraction, ultrasonic extraction, microwave extraction, enzyme-assisted extraction, SFE-CO_2_, etc. (Table 1).

The removal of organic solvents has always been a critical problem in the process of industrial extraction of natural products. At present, the main methods of organic solvent removal are distillation [59,60], acid gas removal [61], chemical oxidation [62], SFE-CO_2_ [63], etc. However, in the process of organic solvent removal, it is often necessary to consider the introduction of new impurities [64]. However, compared with other extraction methods, which relies heavily on organic solvents, SFE-CO_2_ could greatly improve the extraction efficiency by only a small amount of polar solvents. SFE-CO_2_ could even extract small polar components without using organic solvents. Therefore, SFE-CO_2_ still has more advantages than traditional extraction methods, and is greener and environmentally-friendly.

In addition, the traditional natural active extraction method is often cumbersome, which requires repeated extraction and separation process to get the target product. SFE-CO_2_ could get the target product directly by changing the extraction temperature, pressure, and other related factors. This high selectivity makes SFE-CO_2_ more suitable for the extraction of natural products, saving time and raw materials.

Therefore, SFE-CO_2_, with the physical characteristics, high efficiency, energy saving, and high selectivity, is more and more used for the extraction of ginkgo effective components [65].

**Table 1:** The extraction methods and characteristics of active components from *Ginkgo biloba* L.

Methods	Characteristics	References
Solvent extraction	Advantages: simple process, easy industrialization Disadvantages: high energy consumption, high pollution, low efficiency	[66,67,68]
Ultrasonic extraction	Advantages: low energy consumption, mild conditions Disadvantages: difficult to industrialize	[69,70]
Microwave extraction	Advantages: good selectivity, energy saving Disadvantages: easy to damage the active ingredients	[71,72]
Enzyme assisted extraction	Advantages: mild conditions, environmental protection Disadvantages: enzyme activity is easily damaged	[73,74]
Ionic liquid extraction	Advantages: environmental protection, mild conditions, the high recycling rate Disadvantages: toxicological effects unknown, difficult to achieve industrial production	[75,76]
SFE-CO_2_	Advantages: mild conditions, high efficiency, less pollution, high selectivity Disadvantages: high cost, difficult to achieve industrial production	[57,77]

*Ginkgo biloba* contains a variety of bioactive components, including flavonoid [78], ginkgolide [79], proanthocyanidins [65], and polysaccharides [80]. Among them, ginkgo flavones and ginkgolides are the most representative bioactive components. The application of SFE-CO_2_ in the extraction of bioactive components from *ginkgo* will greatly improve the yield and purity of bioactive components, at the same time, reduce the use of organic solvents.

The high efficiency and less pollution have made the SFE-CO_2_ on ginkgo a hotspot that satisfied the demands of the current market and was favored by consumers. Various factors affect the process of SFE-CO_2_ on ginkgo and the productivity of bioactive components, such as extraction pressure, extraction temperature, extraction time, and trainer. Nevertheless, SFE-CO_2_ on the effective components from ginkgo is still in its infancy and much more research is still needed.

## 5. Application on SFE-CO_2_ of Active Components from *Ginkgo biloba* L.

In addition to ginkgo leaves, ginkgo seeds, pollen, root bark, and branch bark could be developed as medicine [34,37,81,82], health food [81,83,84], and cosmetics [81,85]. The content of active ingredients in different parts of ginkgo is quite different. It has been reported that the contents of flavonoids and lactones in ginkgo leaves were higher than those in other parts, while the contents of ginkgolic acid in testa and branches were higher than those others [86]. The various biological activities and the worldwide plentiful resources of ginkgo have made SFE-CO_2_ of ginkgo a promising prospect. At present, the extraction of the effective components of *Ginkgo biloba* by SFE-CO_2_ is mainly concentrated in different parts. The extraction parameters of SFE varied according to the characteristics of active ingredients in different parts of ginkgo (Table 2).

### 5.1. Application of SFE-CO_2_ on Active Components from Ginkgo Leaves

Ginkgo leaves—the parts with the highest content of flavonoids and ginkgolides in *Ginkgo biloba*—have always been the foremost parts for the extraction of effective components of ginkgo. Thus, studies on the extraction of ginkgo by SFE-CO_2_ have primarily focused on its leaves.

#### 5.1.1. The Influence Factors of SFE-CO_2_ on Ginkgo Leaves

Co-solvents were often added to increase the polarity of CO_2_ fluid in the process of extracting flavonoids and lactones from the ginkgo, which could raise the extraction yield of flavonoids and lactones from ginkgo. Flavonoids are phenolic compounds that have many hydroxyl and carbonyl groups in their molecules, and are easy to form special intermolecular forces such as hydrogen bonds with co-solvents. The esters in terpenes also have hydroxyl and carbonyl groups, which are easy to form hydrogen bonds. Zeng et al. [92] investigated the effects of co-solvents on the SFE of ginkgo leaves and found that the addition of co-solvents could effectively improve extraction efficiency. Compared with acetone, ethanol was more likely to form hydrogen bonds with ginkgo flavones, which could make the extraction efficiency raise more. Liu et al. [93] explored the effects of the co-solvent addition method, type, and additional amount as well as flow rate on the SFE of ginkgo leaves. The results indicated that the optimum extraction conditions were as follows: entrainment mode is pre-leaching and dynamic-extraction, extraction pressure 20 MPa, extraction temperature 60 °C, 95% (*v*/*v*) ethanol as co-solvent, the ratio of material to solvent 6:1, and flow rate 10 mL/min. The extraction rate of total flavonol glycosides from ginkgo leaves reached 5.03%. Guo et al. [94] found that co-solvent was the main factor affecting the SFE-CO_2_ process of ginkgolides B from ginkgo leaves, and methanol was the optimal choice of entrainment agent. Guo et al. [95] explored the effect of mixed co-solvent on the SFE-CO_2_ process of ginkgolide B from ginkgo leaves. They mixed methanol and ethanol in the ratio of 9:1 to prepare the co-solvent. The extraction time was 1 h, the extraction pressure was 21 MPa, the extraction temperature was 45 °C, and the amount of co-solvent was 9%. The optimal extraction conditions of ginkgolide B were obtained. The content of ginkgolide B in the extract was up to 0.98%. The above studies implied that when the co-solvents (toxic solvents of methanol and acetone, or non-toxic solvent of ethanol) are used in SFE-CO_2_ process, the elimination of co-solvents after SFE-CO_2_ might be considered. However, there was little research focusing on the removal of co-solvents, which would be worthy of further discussion.

The extraction pressure was the uppermost parameter affecting the extraction yield in the process of SFE-CO_2_ on ginkgo flavonoids, then followed by the extraction temperature. The influence of extraction time and co-solvent on the extraction results was insignificant [87]. The optimum extraction conditions in Zhao’s study were as follows: extraction pressure 35 MPa, extraction temperature 50 °C, extraction time 1.5 h, and 90% (*v*/*v*) ethanol as co-solvent [87].

In addition, temperature is a key factor affecting the extraction of active components from ginkgo. Zhang et al. [96] found that some ginkgolides may decompose with the increase of extraction temperature and time for their unstable structure. At the same time, since the ester affinity of ginkgolides was better, only a small amount of co-solvent was needed. While the polarity of flavonoids was larger, so more co-solvents were needed to achieve a better extraction effect.

In regards to the SFE-CO_2_ process of *Ginkgo biloba* active ingredients, the pretreatment of raw materials also has a great influence on the extraction results. In the pretreatment process, the average particle size of raw materials is an important factor affecting the SFE-CO_2_ of active ingredients. Zhang [88] found that in the extraction process of ginkgo flavones and ginkgolides, the influence of raw material mesh on the extraction results always ranked second. In the process of SFE-CO_2_, pre-soaking of raw materials is also helpful in improving the extraction rate. Zhang et al. [97] optimized the process of SFE-CO_2_ and crystallization of ginkgolide by using ethyl acetate with a solid–liquid ratio of 8.02:1 to improve the extraction rate. In addition, pretreatment methods, such as water content of raw material [98], the cell wall of raw material [99], acidity and alkalization of raw material [100], and packing density, are all important factors affecting the extraction results of SFE-CO_2_ [101]. At present, the extraction of active components from *Ginkgo biloba* leaves by SFE-CO_2_ is not comprehensive enough, and the above pretreatment factors are not involved. Further research is still needed to determine the influence of the above conditions on the extraction results.

#### 5.1.2. Extraction Condition of Active Components from Ginkgo Leaves by SFE-CO_2_

Ginkgo flavonoids possess multiple pharmacological activities, such as antioxidation, anti-cancer, liver protection, etc. [1]. At present, ginkgo flavonoids are mainly obtained from ginkgo leaves, and there are some reports on the extraction of flavonoids from ginkgo seeds, ginkgo pollen, and ginkgo root bark [102]. Jia [103] crushed the fresh ginkgo leaves and added ethanol and enzyme to get the enzymolysis mixture. Then, the mixture was transferred into an SFE unit, CO_2_ was selected as the extractant, beeswax as the co-solvent, the extraction temperature 20–40 °C, the extraction pressure 20–40 MPa, the flow rate 0.10–0.40 mL/min. After extraction for 1–3 h, the extract was absorbed by ethanol, then enriched and eluted by macroporous resin to obtain ginkgo flavonoids with high purity.

Ginkgolides are principally isolated from ginkgo leaves, which are unique components in *Ginkgo biloba*. Highly purified ginkgolides are usually unavailable owing to the production of associated metabolites. Pan et al. [104] used the SFE device to extract the crude extract of ginkgo leaves and crystallize ginkgolides. They optimized the parameters by the single factor method. Extracting at 55 °C for 1.5 h under the condition of 20 MPa extraction pressure could effectively improve the purity of ginkgolide at greater than 85%. Zhang et al. [97,105] optimized the pretreatment of the mention above extraction crystallization method. First, ethyl acetate was used to pre-extract the crude extract of ginkgo leaves. The optimum conditions were as follows: solid–liquid ratio 8.02:1, temperature 77.20 °C, extraction time 4.32 h, and the extraction rate was 92.62%. Combining this optimization method with SFE technology could effectively improve the extraction rate of ginkgolide.

Zhang [88] explored the best extraction process of ginkgolide A, ginkgolide B, and ginkgo flavone from ginkgo leaves. He found that the content of ginkgolide A could reach 7.8 mg/g when the raw material mesh was 60 mesh, the extraction temperature was 50 °C, the extraction pressure was 30 MPa, and 30% ethanol was selected as the co-solvent. The content of ginkgolide B was 17.26 mg/g under the conditions of 60 mesh, 25 MPa extraction pressure, 45 °C extraction temperature, and 100% ethanol co-solvent concentration. Under the conditions of extraction temperature 50 °C, extraction pressure 30 MPa, raw material 40 mesh, and 30% ethanol as co-solvent, the content of flavonoids in the extract could reach 36.17 mg/g.

### 5.2. Application of SFE-CO_2_ on Active Components in Ginkgo Seeds

#### 5.2.1. Extraction of Ginkgo Oil from Ginkgo Seeds by SFE-CO_2_

Ginkgo seeds are rich in a variety of nutrients, with anticancer, antioxidant, free radical scavenging, and other health-giving properties. The fatty acid is one of the main active components in ginkgo seeds, which has the functions of blood pressure reduction, serum lipids decreasing, as well as prevention and treatment of cardiovascular diseases [106]. Zhou et al. [89] optimized the SFE-CO_2_ parameters of ginkgo oil using the response surface methodology, and obtained the best process with 4.46% yield: extraction pressure 29.01 MPa, extraction temperature 40.38 °C, and extraction time 120 min.

#### 5.2.2. Extraction of Ginkgolic Acids from Ginkgo Exotesta by SFE-CO_2_

Ginkgolic acids are the significant active substances in the exotesta of ginkgo. Although the content of ginkgolic acid should be controlled in ginkgo health care products and pharmaceutical preparations, the ginkgo phenolic acid could be collected and used in the fields of medicine and cosmetics due to its anti-inflammatory, antioxidant, and anti-cancer properties. The low polarity and superior solubility in CO_2_ of ginkgolic acid enabled the nonuse of co-solvent in SFE-CO_2_. Yin et al. [90] found that the extraction of ginkgolic acid from ginkgo exotesta by SFE-CO_2_ at the pressure of 30 MPa, the temperature of 45 °C, and CO_2_ flow rate of 2 L/min for 6 h could obtain a better yield. Compared with the methanol reflux method, the SFE-CO_2_ method could extract a higher yield and higher purity of ginkgolic acid from ginkgo exotesta [107].

### 5.3. Application of SFE-CO_2_ of Effective Components from Ginkgo Pollen

Compared with ginkgo leaves, the flavonoid-rich ginkgo pollen contains more protein, amino acids, and other nutrient contents [102,108,109]. However, only a few studies were about the chemical constituents and pharmacological activities of ginkgo pollen, as well as the SFE-CO_2_ on ginkgo pollen. Ginkgo pollen is rich in the functional factor vitamin E, which has the effects of scavenging free radicals, anti-aging, and prolonging life [34]. Qiu et al. [110] found that ginkgo pollen contained 4.37 times the content of flavonoids in ginkgo leaves by using DPPH-HPLC-PAD and HPLC-ESI-MS^2^ methods. In ginkgo pollen, 96.71% of the aglycones of the flavonoid glycosides were kaempferol, while the main aglycones in leaves were quercetin. The main antioxidant component in pollen was flavonoid glycosides. They also proved that the ethyl acetate extract of ginkgo pollen crude extract had the best DPPH free radical scavenging activity (IC_50_ was 0.46 mg/mL), and kaempferol, the main flavonoid compound in this component, had the best DPPH free radical scavenging activity (IC_50_ was 0.017 mg/mL) [111]. Feng et al. [91] used wall-broken ginkgo pollen as raw material, gelatin, and starch as adhesive, and use SFE-CO_2_ to extract bioactive components from ginkgo pollen. The extraction temperature was 35–65 °C, the extraction pressure was 18–35 MPa, and the CO_2_ flow rate was 10–30 L/h. They removed the adhesive from the extract to get the ginkgo pollen extract. The experimental studies on animals and humans showed that ginkgo pollen extract could effectively improve memory, which was conducive to the development of health-giving food and medical applications.

There are few studies on SFE-CO_2_ in regards to the active ingredients from ginkgo pollen. The nutrient-rich ginkgo pollen has a good effect on scavenging free radicals and improving memory [111], which makes it promising to apply SFE-CO_2_ in ginkgo pollen. Consequently, there is a need for more research on SFE-CO_2_ of ginkgo pollen, to make ginkgo pollen extract more green and safe, and more easily made into medicine and health care products. Researchers should consider using SFE-CO_2_ to extract nutrients from ginkgo pollen in future studies.

### 5.4. Application of SFE-CO_2_ of Effective Components from Branch Bark and Root Bark of Ginkgo

*Ginkgo biloba* has been studied due to its bioactive ingredients in leaves, seeds, pollen, branch bark, and root bark. Among these, ginkgo leaves have been studied more extensively due to their availability and high content of active ingredients. Although studies have shown that ginkgo root bark, branch bark, and other parts contain ginkgo flavones, ginkgolides, and other active components [112,113], at present, there is no relevant research on SFE-CO_2_, except for several reports on the active components from root bark and branch bark of *Ginkgo biloba* by organic solvent extraction. This may be because the root and branch bark of ginkgo are relatively difficult to obtain compared to ginkgo leaves, the damage to the ginkgo tree itself is greater, and the content of effective components is lower.

## 6. Discussion

From studying SFE-CO_2_ of different parts of ginkgo (above), one could see that the extraction conditions are often different for different target products. For ginkgo flavones and ginkgolides extraction, SFE-CO_2_ is often concentrated in ginkgo leaves. The results showed that the optimum extraction conditions were as follows: 40 mesh sieve in ginkgo leaves, extraction temperature 20–50 °C, extraction pressure 20–40 MPa. The extraction of ginkgolides was usually carried out in a 60 mesh sieve of ginkgo leaves. The extraction temperature was 45–55 °C and the pressure was 25–35 MPa.

Although Zhang et al. [96] found that the extraction rate of ginkgolides decreased with the increase of temperature, it was also reported that ginkgolides can remain stable at 100 °C [114]. The above SFE-CO_2_ extraction temperature of ginkgolides did not reach 60 °C. Therefore, the increase of temperature has little effect on the extraction of ginkgolides by SFE-CO_2_. Ginkgo flavones and ginkgolides belong to high polar components. To obtain a better extraction effect, the ethanol with a higher polarity, which is easy to form a hydrogen bond with them, is generally selected as the co-solvent. However, the experimenters chose different concentrations of ethanol, which is difficult to summarize.

Ginkgolic acid and ginkgo oil (different from ginkgo flavone and ginkgolide) belong to small polar components, which could obtain better extraction effects without using co-solvents. The extraction of ginkgo oil and ginkgolic acid by SFE-CO_2_ was concentrated in ginkgo seeds. In the SFE-CO_2_ of ginkgo seed active components listed above, it could be seen that the conditions of ginkgolic acid and ginkgo oil are similar.

In addition, SFE-CO_2_ has a fractionation effect; it could also remove some products from ginkgo without introducing any solvent. This method could be applied to the removal of ginkgolic acid in ginkgo tea. It not only maintains the medicinal components of ginkgo tea, but also improves the quality of ginkgo tea products.

At present, there are few studies on the extraction of ginkgo pollen by SFE-CO_2_, and there is only one related report. The extraction temperature was 35–65 °C, the extraction pressure was 18–35 MPa, and the CO_2_ flow rate was 10–30 L/h [91]. At present, there is no relevant research on the extraction of ginkgo root bark and branch bark by SFE-CO_2_.

The amount of organic solvent introduced by SFE-CO_2_ is far less than that of the non-SFE-CO_2_ extraction method, although polar solvent is introduced into the extraction process of ginkgo flavones and ginkgolides by SFE-CO_2_. In addition, methanol (boiling point: 64.7 °C) and ethanol (boiling point: 78.4 °C) are commonly used as organic solvents in SFE-CO_2_ extraction of ginkgo flavones and ginkgolides. It has been reported that ginkgo flavones have good stability below 80 °C and ginkgolides have good stability below 100 °C [114,115]. Therefore, the removal of organic solvents by vacuum distillation had little effect on the flavonoids and ginkgolides.

However, the current research on the SFE-CO_2_ of active components from ginkgo is not comprehensive enough; the current research almost does not involve extraction factors, such as raw material mesh, water content, pH, etc. Moreover, there are few reports on the extraction of active components from ginkgo pollen, root bark, and branch bark by SFE-CO_2_. Therefore, more experimental data in the field of SFE-CO_2_ of ginkgo active ingredients are needed. Up until now, the extraction of active components from *ginkgo* by SFE-CO_2_ is still in the laboratory scale stage, so we still have a long way to achieve industrial-scale applications. However, SFE-CO_2_ will still be the most promising method to extract active components of *ginkgo* because of its advantages, e.g., high extraction amount, less introduction of organic solvents, and high selectivity. Therefore, SFE-CO_2_ extraction of *Ginkgo biloba* active ingredients, in the future, will have vast potential when applied in various industries (i.e., medical, food, cosmetics, etc.).

## 7. Conclusions

Compared with the traditional organic solvent extraction method, SFE-CO_2_ technology, as a new, environmentally-friendly green extraction process, has the advantages of higher yield and efficiency, energy-saving, short extraction time, higher concentration of active ingredients, superior pharmacological effect, and lower toxicity. With further research, SFE-CO_2_ will be applied in a broader field, especially in the field of traditional Chinese medicine, which could be applied to the development of the extraction and separation of traditional Chinese medicine.

*Ginkgo biloba* possesses excellent biological activities and is widely used in many fields. The application of SFE-CO_2_ on ginkgo could shorten the extraction time and lead to a higher yield. With the relatively lower temperature of SFE-CO_2_, the structure of active components in ginkgo extract became more stable, and the content and purity are more promising. However, industrial research on the extraction of the effective components of ginkgo by SFE-CO_2_ is still relatively weak. It is believed that, with the deepening of research and the development of SFE technology on ginkgo, it will be extensively applied in certain industries, including the medical, food, cosmetics fields.

## Figures and Tables

**Table 2:** Application of SFE-CO_2_ to extract active components from different parts of *Ginkgo biloba* L.

Part	Main Components	SFE Parameters	References
Ginkgo leaf	Ginkgo flavone	Temperature: 50 °C, Pressure: 35 MPa, Co-solvents: ethanol 90% (*v*/*v*), Extraction time: 1.5 h	[87]
	Ginkgolide A	Temperature: 50 °C, Pressure: 30 MPa, Co-solvents: ethanol 30% (*v*/*v*), Raw material mesh: 60 mesh Yield: 7.8 mg/g	[88]
	Ginkgolide B	Temperature: 45 °C, Pressure: 25 MPa, Co-solvents: ethanol 100% (*v*/*v*), Raw material mesh: 60 mesh Yield: 17.26 mg/g	[88]
Ginkgo seed	Ginkgo oil	Temperature: 40.38 °C, Pressure: 29. 01 MPa Extraction time: 2 h Yield: 4.46%	[89]
	Ginkgolic acid	Temperature: 45 °C, Pressure: 30 MPa, CO_2_ flow rate: 2 L/min, Extraction time: 1.5 h	[90]
Ginkgo pollen	Total extract of ginkgo Pollen	Temperature: 35–65 °C, Pressure: 18–35 MPa, CO_2_ flow rate: 10–30 L/h Raw material mesh: 20–40 mesh Adhesive: gelatin and starch	[91]

## Data Availability

Not applicable.
